# Prediction of premyopia and myopia in Chinese preschool children: a longitudinal cohort

**DOI:** 10.1186/s12886-021-02045-8

**Published:** 2021-07-21

**Authors:** Lei Liu, Rui Li, Dan Huang, Xiao Lin, Hui Zhu, Yue Wang, Xiaoyan Zhao, Xiaohan Zhang, Hu Liu

**Affiliations:** 1 Department of Ophthalmology, The First Affiliated Hospital with Nanjing Medical University, 300 Guangzhou Road, Nanjing, 210029 China; 2 Department of Child Healthcare, The First Affiliated Hospital with Nanjing Medical University, Nanjing, China; 3 University of Houston, College of Optometry, Houston, TX USA; 4 Department of Ophthalmology, The Affiliated Changzhou No.2 People’s Hospital of Nanjing Medical University, Changzhou, China; 5 Department of Ophthalmology, Wuxi Children’s Hospital, Wuxi, China

**Keywords:** Premyopia, Myopia, Prediction, Preschool children

## Abstract

**Backgrounds:**

Myopia has become a global public health problem. Children with early onset of myopia are at particular risk of complications associated with myopia. Younger children and children with greater initial myopic refractive errors are at a greater risk of myopia progression. Therefore, it is essential to identify subjects at high risk of developing myopia to facilitate myopia prevention in the early stage, especially during the preschool period. The purpose of this study was to determine whether premyopia and myopia in preschool children can be predicted by easily obtainable parameters.

**Methods:**

Data was collected in a population-based cohort. Comprehensive examinations included height, weight, refraction, axial length (AL), and corneal radius of curvature (CR), with a follow-up of 2 years. Parental myopia history was obtained from a questionnaire. Myopia was defined as spherical equivalent (SE) ≤ − 0.50 D. Premyopia was defined as − 0.50 D < SE ≤ + 0.75 D. Multivariate linear regression models were fitted to determine the associations between these parameters at baseline and future SE. To predict premyopia and myopia, Cox proportional hazard regression analysis coupled with a nomogram was used.

**Results:**

A total of 830 children (433 boys and 397 girls) were included (40.83 ± 3.43 months old at baseline). A significantly negative relationship was observed in the multivariate analysis between baseline AL, AL/CR, two myopic parents, and the future SE after adjusting for age and gender (coefficient = − 0.291, coefficient = − 5.791, coefficient = − 0.273, respectively, both *p* <  0.001). Higher baseline AL, AL/CR (hazard ratio (HR) = 4.916, HR = 2.979, respectively, comparing the top quartile with the bottom quartile, both *p* <  0.001) and two myopic parents (HR = 1.756, compared to no myopic parents, *p* = 0.001) were associated with a higher risk of future onset of premyopia. From the nomogram, AL/CR was found to have the most enormous effect on survival. Different baseline AL and AL/CR values (both Log Rank *p* <  0.001) had different survival curves.

**Conclusions:**

AL and AL/CR could be used as obtainable indicators for identifying subjects at high risk of developing premyopia and myopia in young preschool children.

## Background

Over the past decade, myopia has become a global public health problem. The myopia boom is growing globally, and 49.8% of the world population is predicted to be myopic, and 9.8% of the world population is predicted to be high myopic in 2050 [1]. The prevalence of myopia for preschool children (younger than 7 years old) has been reported to be greater than 5% [2, 3]. Children with early onset of myopia are at particular risk of complications associated with myopia. Myopia progression over time could result in high myopia, which is related to some irreversible blinding complications, such as retinal detachment, myopic macular degeneration, and glaucoma [4]. Also, younger children and children with greater initial myopic refractive errors are at a greater risk of myopia progression [5, 6]. Therefore, it is essential to identify subjects that are at high risk of developing myopia to facilitate myopia prevention in the early stage, especially during the preschool period. As such, International Myopia Institute (IMI) recently introduces the concept of premyopia, which is defined as SE between − 0.50 D and + 0.75 D, to guide research on myopia prevention [7]. However, factors for predicting premyopia or myopia onset have not been reported in preschool children.

Several cohort studies have evaluated factors associated with myopia incidence and progression rate among children. Initial spherical equivalent (SE) is considered to be the single best predictive factor for the school children [8, 9]. However, the risk factors for the prediction of myopia onset have not been reported in preschool children. Previous studies showed that due to the influence of accommodation, noncycloplegic assessment of refractive errors in children overestimates myopia and results in a high error rate for emmetropic and hyperopic refractive errors [10, 11]. However, measuring SE by cycloplegic refraction in preschool children is limited by poor cooperation and long examination time. Thus, alternative myopic indicators that are easily obtainable can merit preventative interventions for myopia. The visual system keeps the refractive state in the normal range through balancing changes in the axial length (AL) and the refractive components, including the cornea and the lens. When the compensation mechanism of emmetropization between refractive parameters is out of balance, there is a tendency to myopia onset [12, 13]. According to a cross-sectional study, long AL is a risk factor for early-onset myopia in school children [14]. Also, some studies on myopia onset have shown stronger correlations between myopia and the ratio AL/corneal radius of curvature (CR) than with AL or CR alone [15, 16]. AL is usually measured using partial coherence interferometry (PCI) and is testable in preschool children [17]. According to cross-sectional studies, AL, AL/CR have been found to highly correlate with noncycloplegic SE in preschool children [16, 18].

We hypothesized that both premyopia and myopia in preschool children could be predicted by ocular biometric parameters, such as AL and AL/CR. In the present study, we aimed to examine the associations between AL and AL/CR at baseline and future SE in preschool children and identify factors for predicting premyopia and myopia.

## Methods

### Study population

The Nanjing Eye Study (NES) is a population-based cohort study aiming to longitudinally observe the onset and progression of childhood ocular diseases in eastern China [19]. As described previously, in Yuhuatai District, Nanjing, China, all children born between September 2011 and August 2012 and entering kindergarten in Yuhuatai District were invited to participate in NES and undergo comprehensive examinations. Baseline data was obtained in 2015 when the children were between 36 and 48 months old, and, follow-up data was acquired in 2016 and 2017 with the mean interval between each year of 12 ± 1 month.

The NES study was approved by the Ethics Committee of the First Affiliated Hospital with Nanjing Medical University and followed the tenets of the Declaration of Helsinki. Written informed consent was obtained from the parents or legal guardians of all participants. Verbal consent was obtained from all children right before the examination. The examinations were supervised by attendant teachers or guardians.

### Examinations and questionnaire

A comprehensive examination of all participants was performed by a team composed of 4 junior ophthalmologists, 2 senior ophthalmologists, and 4 optometrists using similar protocols as described elsewhere [20]. Seniors reviewed examinations performed by juniors. Basic information, including name, gender, and birth date, was obtained from each kindergarten’s principal and was checked during the examination. Basic examinations, including anthropometric parameters, visual acuity, anterior segment, and fundus examination, refraction without cycloplegia, stereoacuity test, ocular alignment and motility, and ocular biometric parameters, were performed in the setting of each kindergarten. Children with suspected or confirmed eye problems were referred to senior ophthalmologists and underwent further examinations.

Ocular biometric parameters, including AL and CR, were measured by IOL Master (Carl Zeiss Meditec, Jena, Germany; V5.5.0.0062) before cycloplegia with standard room lighting. The right eye was tested first. All ocular biometric parameters, including AL, the greatest corneal radius of curvature (CR1), and the lowest corneal radius of curvature (CR2), were performed five times. The mean values were used for analysis.

Cycloplegic refraction was performed after cycloplegia using table-mounted autorefraction (Cannon R-F10, Tokyo, Japan) and retinoscopy for children with suspected or confirmed eye problems in baseline grade and children whose guardians signed the informed consent of cycloplegia in both middle and graduation grades. Two drops of topical 1.0% cyclopentolate (Cyclogyl, Alcon Pharmaceuticals) were administered to each eye at a 5-min interval. After 15 min, a third drop of cyclopentolate was applied if the pupil size was < 6 mm or the pupillary light reflex was still present.

Height in centimeters was measured without shoes. Weight in kilograms was measured using a standard weighing machine, which was calibrated before the examination.

A comprehensive questionnaire was self-administered by legal guardians at baseline, including parental myopia, outdoor time and near activities time [21, 22].

### Definitions

SE was calculated as spherical power plus half of the cylindrical power. In accordance with the definition in IMI [7], myopia was defined as SE ≤ − 0.50 D, and premyopia was defined as − 0.50 D < SE ≤ + 0.75 D.

CR was calculated as the average of CR1 and CR2. Corneal power (CP) in diopters was calculated using the formula corneal power (D) = 0.3375*1000/CR (mm); the AL-to-CR ratio (AL/CR) was calculated as AL in millimeters divided by CR in millimeters.

Parental myopia represented the number of myopic parents, including zero, one, and two myopic parents.

### Statistical analysis

Data analysis was performed using the IBM Statistical Package for the Social Sciences program V13.0 (SPSS, Chicago, IL, USA) and R software (version 4.0.3, https://www.r-project.org/). Descriptive statistics were presented using mean ± standard deviation (SD) for continuous variables and percentages for the categorical variables. *P* value < 0.05 was considered statistically significant. All confidence intervals were 95%.

Analysis of variance (ANOVA), independent-samples t-test, and Pearson χ^2^ test were applied to compare differences between groups. Univariate linear regression analyses were performed to assess the association of baseline factors, including age in month, gender, height, weight, CP, AL, AL/CR, and parental myopia, with SE in the graduation grade in the kindergarten. A multivariate linear regression model was then fitted with age, gender, height, weight, and other variables with *p* <  0.1 from univariate analyses. To examine factors associated with incident premyopia and myopia, Cox proportional hazard regression analysis was conducted on participants who were not in the stage of myopia or premyopia at baseline. For comparing students by height, weight, CP, AL, and AL/CR, a reference group was defined using participants in the bottom quartile (< the 25th percentile) of the entire cohort. ROC curve and Hosmer-Lemeshow test were fitted to examine the discrimination and calibration of the Cox model. The candidate predictors with significant *P* values were then incorporated into the nomogram. AL and CR between the right and left were highly correlated (Pearson correlation coefficient = 0.982, Pearson correlation coefficient = 0.971, respectively, both *p* <  0.001). Therefore, the data from the right eye were used for analysis.

## Results

Among the total 1491 children who cooperated with the IOL Master and had a complete data set at baseline, 90 (6.04%) children had strabismus, amblyopia, or glasses for refractive error, thus leaving 1401 children.

In total, 830 children (433 boys and 397 girls), whose guardians filled in the questionnaire and signed the informed consent about taking cycloplegic examinations, were included in the study for myopia prediction factors. There was no statistically significant difference between 830 included children and 571 excluded children in gender (*p* = 0.891), age (*p* = 0.446), height (*p* = 0.365), weight (*p* = 0.566), CP (*p* = 0.803), AL (*p* = 0.240) and AL/CR (*p* = 0.246) (Table 1). Among the 830 children, 79 (9.52%) and 109 (13.13%) children were lost during follow-up in middle and graduation grades, respectively. In addition, 130 (15.66%) and 95 (11.45%) children became premyopic or myopic in middle and graduation grades, respectively.

**Table 1:** Characteristics at baseline between included and excluded children

Baseline Characteristics	Included Children (***N*** = 830)	Excluded Children (***N*** = 571)	P^*^ Value
Boy: Girl	433:397	300:271	0.891
Age (month)	40.83 ± 3.43	40.97 ± 3.31	0.446
Height (cm)	100.25 ± 4.37	100.46 ± 4.32	0.365
Weight (kg)	15.88 ± 2.43	15.80 ± 2.24	0.566
CP (diopter)	43.44 ± 1.42	43.46 ± 1.43	0.803
AL (mm)	21.90 ± 0.65	21.86 ± 0.62	0.240
AL/CR	2.82 ± 0.06	2.81 ± 0.06	0.246

*Abbreviations*: *CP* corneal power, *AL* axial length, *CR* corneal radius of curvature

^*^ Determined using independent-samples t-test and Pearson χ^2^ test

Table 2 showed characteristics of children in 2 years’ follow-up. The mean age was 40.83 ± 3.43 months at baseline (2015) and 66.81 ± 3.33 months at graduation (2017). Age-related increase was statistically significant in AL and AL/CR (21.90 ± 0.65 mm and 2.82 ± 0.06 at baseline, compared to 22.48 ± 0.70 mm and 2.87 ± 0.13 at graduation; both *p* <  0.001), while CP decreased with age (43.44 ± 1.42 D at baseline, compared to 43.15 ± 1.41 D at graduation, *p* <  0.001).

**Table 2:** Characteristics of preschool children by 2 years’ follow-up

Characteristics	Age (months)			P^*^Value
	36–48 (Baseline, 2015)	48–60 (Middle, 2016)	60–72 (Graduation, 2017)	
Age (month)	40.83 ± 3.43	54.89 ± 3.44	66.81 ± 3.33	< **0.001**
Height (cm)	100.25 ± 4.37	107.74 ± 5.51	114.40 ± 5.94	< **0.001**
Weight (kg)	15.88 ± 2.43	18.41 ± 2.77	20.95 ± 3.93	< **0.001**
CP (diopter)	43.44 ± 1.42	43.18 ± 1.41	43.15 ± 1.41	< **0.001**
AL (mm)	21.90 ± 0.65	22.26 ± 0.69	22.48 ± 0.70	< **0.001**
AL/CR	2.82 ± 0.06	2.84 ± 0.13	2.87 ± 0.13	< **0.001**

Bold type indicated statistical significance

*Abbreviations*: *CP* corneal power, *AL* axial length, *CR* corneal radius of curvature

^*^ Determined using analysis of variance (ANOVA)

In the univariate models, of all studied variables, AL, AL/CR (baseline grade) and two parental myopia were consistently correlated to the future SE (graduation grade) (coefficient = − 0.309; coefficient = − 6.341; coefficient = − 0.505, respectively, both *p* <  0.001). In the multivariate model, significant negative relationships were observed between AL, AL/CR, and SE after adjusting for age and gender. There were 0.30 D and 5.79 D shifts towards myopia with every 1 unit increase in the AL and AL/CR, respectively. Compared with one- and no-myopic-parent, having two myopic parents was associated with refraction (coefficient = − 0.273, *p* <  0.001). However, height and weight were not correlated with refraction (Table 3).

**Table 3:** Association between ocular parameters and anthropometry at baseline with SE at graduation

Characteristics	Univariate Analysis		Multivariate Analysis		Standardized coefficient
	Coefficient (95% CI)	P^*^ value	Coefficient (95% CI)	P^*^ value	
Gender	0.080 (−0.039 to 0.198)	0.186	−0.171 (−0.282 to − 0.060)	**0.003**	− 0.114
Age (month)	0.014 (−0.003 to 0.031)	0.108	0.029 (0.013 to 0.045)	**< 0.001**	0.132
Height (cm)	−0.005 (− 0.019 to 0.008)	0.429	0.003 (− 0.01$ to 0.020)	0.697	0.020
Weight (kg)	−0.022 (− 0.046 to 0.001)	0.063	− 0.005 (− 0.033 to 0.023)	0.727	−0.017
No. of Parental Myopia					
0	NA	NA	NA	NA	NA
1	−0.128 (−0.265 to − 0.010)	0.069	− 0.063 (− 0.181 to − 0.055)	0.295	−0.036
2	−0.505 (− 0.660 to − 0.350)	**0.000**	−0.273 (− 0.410 to − 0.136)	**< 0.001**	−0.139
CP (diopter)	−0.036 (− 0.078 to 0.005)	0.083	NA	NA	NA
AL (mm)	−0.309 (− 0.398 to − 0.221)	< **0.001**	−0.291 (− 0.381 to − 0.202)	< **0.001**	−0.250
AL/CR	−6.341 (−7.231 to −5.451)	< **0.001**	−5.791 (−6.678 to −4.904)	< **0.001**	− 0.448

Bold type indicated statistical significance

*Abbreviations*: *CI* confidence interval, *NA* not applicable, *CP* corneal power, *AL* axial length, *CR* corneal radius of curvature

^*^Determined using linear regression analysis

**Fig. 1:**
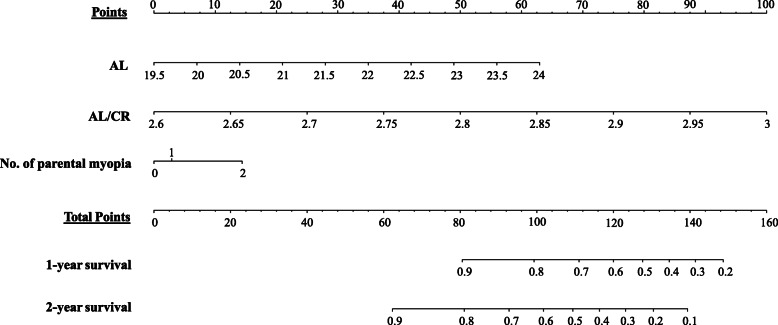
A nomogram for predicting 1- and 2-year overall survival of premyopia and myopia. Instructions for using the nomogram. Draw a perpendicular line upward to determine the score of each variable in the points axis. Sum up the number of points for all variables then find the location in the total points axis. Draw a perpendicular line down to the survival axes to determine 1-, 2-year survival probabilities. For classified variables, 0 = no myopic parents, 1 = one myopic parent, 2 = two myopic parents. Abbreviations: AL, axial length; CR, corneal radius of curvature

Cox proportional hazard regression analysis for predicting myopia or premyopia in the cohort is shown in Table 4, which excluded two children of myopia and 72 children of premyopia at baseline. After adjusting for other covariates, higher baseline AL (hazard ratio (HR) = 2.875, for the middle quartile compared with the bottom quartile; HR = 4.916, for the top quartile compared with the bottom quartile; both *p* <  0.001), higher baseline AL/CR (HR = 1.702, for the middle quartile compared with the bottom quartile, *p* = 0.022; HR = 2.979, for the top quartile compared with the bottom quartile, *p* <  0.001) and two myopic parents (HR = 1.756, compared with no myopic parents, *p* = 0.001) were statistically significantly associated with incident myopia and premyopia in future. However, baseline height (*p* = 0.093), weight (*p* = 0.652), CP (*p* = 0.259), and only one myopic parent (*p* = 0.590) had no significance. A nomogram incorporating the above three independent predictive factors with significant *P* values was built (Fig. 1). AL, AL/CR, and number of myopic parents each result in points from 0 to 63 (AL), 0 to 100 (AL/CR), and 0 to 25 (number of myopic parents), and the total points range from 0 to 160. Individual risk score is obtained by summing each separate risk factor. By the sum of the risk score, we might estimate the probability of 1-year and 2-year survival, which was the probability of not developing pre- or myopia. The higher the total score, the lower the survival rate.

**Table 4:** Factors associated with incident myopia and premyopia

Baseline Features^a^	Adjusted Hazard Ratio^b^	95% Confidence Interval	P^*^ Value (for Trend)
Sex			
Male	Reference		
Female	1.073	0.806–1.429	0.628
Height (cm)			
< 97 (25th Percentile)	Reference		0.093
97–103.2	0.654	0.444–0.963	**0.031**
> 103.2 (75th Percentile)	0.657	0.406–1.064	0.088
Weight (kg)			
< 14.1 (25th Percentile)	Reference		0.652
14.1–17.1	1.166	0.782–1.739	0.450
> 17.1 (75th Percentile)	1.257	0.769–2.054	0.363
CP (diopter)			
< 42.50 (25th Percentile)	Reference		0.259
42.50–44.35	1.383	0.911–2.102	0.128
> 44.35 (75th Percentile)	1.629	0.877–3.025	0.122
AL (mm)			
< 21.42 (25th Percentile)	Reference		< **0.001**
21.42–22.33	2.875	1.706–4.842	< **0.001**
> 22.33 (75th Percentile)	4.916	2.537–9.527	< **0.001**
AL/CR			
< 2.77 (25th Percentile)	Reference		< **0.001**
2.77–2.85	1.702	1.078–2.688	**0.022**
> 2.85 (75th Percentile)	2.979	1.760–5.045	< **0.001**
No. of parental myopia			
0	Reference		**0.005**
1	1.093	0.791–1.510	0.590
2	1.756	1.250–2.467	**0.001**

Bold type indicated statistical significance

*Abbreviations*: *CP* corneal power, *AL* axial length, *CR* corneal radius of curvature

^a^ Percentiles correspond to baseline examination values for all preschool children

^b^ Adjusted hazard ratios for each group adjusting for all other covariates

*Determined using cox proportional hazard regression analysis

**Fig. 2:**
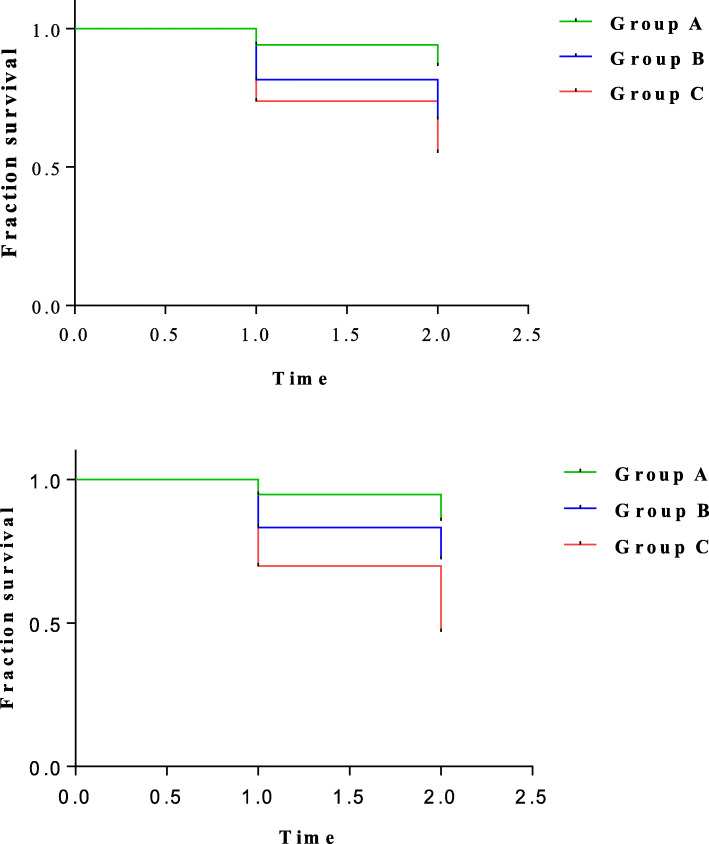
Survival curves for incident myopia and premyopia in different AL and AL/CR groups. Top: Group **A**: AL < 21.42 mm (25th Percentile), Group **B**: AL = 21.42 mm to 22.33 mm, Group **C**: AL > 22.33 mm (75th Percentile). Bottom: Group **A**: AL/CR < 2.77 (25th Percentile), Group **B**: AL/CR = 2.77 to 2.85, Group **C**: AL/CR > 2.85 (75th Percentile)

The survival curves for incident myopia and premyopia in different AL and AL/CR groups are shown in Fig. 2. Different baseline AL and AL/CR (both Log Rank *p* <  0.001) values had different survival curves. The incidence of myopia and premyopia were highest when baseline AL > 22.33 mm (75th percentile) and AL/CR > 2.85 (75th percentile) compared to the other two groups.

## Discussion

In the present study, we use ocular biometric parameters and physical parameters to predict premyopia and myopia in a cohort of preschool children in eastern China. Higher baseline AL and AL/CR are related to more myopic SE 2 years later. AL and AL/CR are the high-risk indicators of the incident premyopia and myopia among preschool children. We conclude that AL and AL/CR examinations might help identify children susceptible to myopia prospectively. This is the first study on predicting premyopia and myopia in preschool children to the best of our knowledge.

The refractive development of human eyes is a dynamic process that ocular biometric parameters change from birth, and the refractive state changes accordingly [12]. The visual system keeps the refractive state in normal range by balancing changes in the AL and other refractive components, including the cornea and the lens [13, 23]. Previous studies on refractive development suggest that over the first year or two after birth, AL elongation is coordinated with corneal flattening, and, after that, CR is relatively stable, but AL elongation exists for a long time [13]. Our observation is consistent with this pattern of refractive development. In our study, the age-related increase was statistically significant in both AL and AL/CR, consistent with the Shenzhen cross-sectional study, which showed AL and AL/CR increased from 3 to 6 years of age (AL from 22.19 ± 0.65 mm to 22.63 ± 0.63 mm, AL/CR from 2.84 ± 0.06 to 2.91 ± 0.07) [18]. Meanwhile, our study found a flattening in CR over 2 years, consistent with previous research on the refractive development of 3- to 6-year-old children in Shanghai [24]. Nonetheless, earlier studies for school-aged children suggested CR varies little from ages 6 to 14 years [25, 26]. The difference may be due to the study design and age of the included subjects. We speculate that the flattened cornea is to compensate for the elongation of the axial length during the preschool period.

Over the past decade, myopia’s prevalence has increased quickly, whereas the age of myopia onset has decreased. A significant increase in myopia has been noted in Hong Kong preschool and school children. The prevalence of myopia was 6.3% in preschoolers. Meanwhile, the prevalence of myopia reached 12.7% in schoolchildren aged 6 years [27, 28]. The Singapore Cohort of the Risk Factors for Myopia (SCORM) study found that the onset age of myopia or myopia progression duration were the strongest predictors of high myopia in their later life [29]. Sara et al. [30] found that a younger age of myopia onset was associated with more rapid myopia progression. It has been reported that early achievement of emmetropia is a risk factor for subsequent progression to myopia [23]. Noteworthily, previous longitudinal studies of refractive development in school children found that most forms of myopia progression have variable periods. Particularly, before the onset of myopia, eyes showed an accelerated axial elongation pattern and refractive error for several years. The acceleration of axial elongation is faster than the one that occurs after the onset of myopia [31, 32]. These results indicated the importance of myopia prevention in the stage just before the onset of myopia. IMI has made it clear that premyopia is a refractive state of an eye of ≤ + 0.75 D and > − 0.50 D in children, which could be considered as a framework for research on myopia prevention.

Previous studies on myopia prediction mainly focused on school-age children. The National Eye Institute-funded Collaborative Longitudinal Evaluation of Ethnicity and Refractive Error (CLEERE) study assessed the ability of 13 candidate risk factors to predict the onset of myopia and found that SE was the single best predictive factor [33]. Therefore, trials for myopia prevention should target children with low hyperopia. Limited by poor cooperation and low follow-up rate of young children, few studies have assessed the probability of myopia in preschool children through a longitudinal observation. Moreover, it is more difficult to collect cycloplegic refraction data than collect ocular biometric parameter values in preschoolers, suggesting that ocular biometric parameter values might be a more realistic alternative data source for myopia prediction in preschoolers. AL, AL/CR have been found to highly correlate with noncycloplegic SE in preschool children, according to cross-sectional studies [16, 18]. Also, a 1.5-year follow-up among school-age children in China indicated that although there were confounding factors, the use of the current AL/CR as a precursor indicator for myopia onset is undeniable [34]. Therefore, we hypothesized that early myopia and premyopia could be predicted by ocular biometric parameters, such as AL and AL/CR, and other risk factors.

In the present cohort, we found that among all studied variables, AL and AL/CR (baseline grade) were consistently associated with the future SE (graduation grade) after adjusting for age and gender. Therefore, baseline AL and AL/CR could be used to monitor refractive development and the tendency to develop myopia. Given the relatively low prevalence of myopia in preschoolers, we selected those who were in the stages of premyopia or myopia as a study population and took myopia and premyopia as occurrence events in the prediction model. We found that higher AL value (AL > 21.42 mm) and higher AL/CR (AL/CR > 2.77) at baseline were the independent factors most strongly associated with incident myopia and premyopia. Using the nomogram as a visual and graphical prediction tool, AL/CR was found to have the most enormous effect on survival. Nonetheless, a large external validation cohort is needed to validate the nomogram. Thus, a systematic, prospective evaluation of our model in a larger patient population is warranted.

Consistent with other longitudinal studies, we also found a higher risk of myopia in children with two myopic parents compared with those in one- and no-myopic-parent families [35–37]. However, we did not find a statistically significant difference between children having only one myopic parent and children not having a myopic parent in the risk of myopia onset. The nonsignificant difference between children with or without a myopic parent may be explained that refractive error development is affected by both the environment and genetic factors. Due to the lack of complete questionnaire data in baseline and middle grades, we retrospectively checked the questionnaire from graduation grades. We found that outdoor time and near activities time were 2.25 h/day and 4.32 h/day among children who were pre- or myopia, 2.30 h/day and 4.21 h/day among children who were nonmyopic. There were no statistically significant between difference two groups of children (*P* = 0.586, *P* = 0.498). The possible explanation could be questionnaires with poor response rates and poor quality or the less educational time and more similar life pattern during preschool. In the GUSTO birth cohort study, genetic factors are suggested to have a greater contribution to early development of refractive error compared to environmental factors in preschool children [14].

We found no correlation between baseline physical parameters and future SE, which is consistent with the Growing Up in Singapore Towards Healthy Outcomes (GUSTO) birth cohort study in Singapore [14]. Conversely, Ye S et al. found that personal anthropometry values, such as height and weight, remained independently related to refraction among schoolchildren aged 6–15 years old in Tianjin, China [38]. We infer that the absence of a relationship between anthropometric measures and SE may be due to the difference in the study population that varies in corneal and lens powers, which compensate for AL growth, although the eye grows in harmony with the body.

This study’s strengths include large-scale longitudinal data from preschool children in a particular period of eye development and the objective measure of refraction with complete cycloplegia. While, there were also some limitations. Firstly, we failed to get cycloplegic refraction in all children at baseline, but we found no significant differences in the baseline parameters between children included and excluded. Secondly, we did not obtain the data of lens power, as an IOL Master could not measure lens power directly. While, some studies indicated that lens power also played an essential role in refractive development [32]. Thirdly, our follow-up time was a fixed node, and we were unable to determine the exact time of the onset of myopia or premyopia. Fourthly, we could not evaluate environmental factors’ influence in the present analysis due to lack of complete questionnaire data in baseline and middle grades about environmental factors, including near activities and outdoor times.

Nonetheless, our study determined easier predictive factors of myopia than traditional cycloplegic refraction in preschoolers despite limitations. Based on these findings, monitoring the AL/CR changes should be informative in identifying children at risk for myopia. Children with higher risk should be provided with preventive advice and monitored closely for the onset of myopia so that anti-myopia therapies can be applied in time [39, 40].

## Conclusions

In conclusion, AL and AL/CR showed an age-related increase from 3- to 6-year-old. Premyopia and myopia could be predicted using baseline AL and AL/CR in preschool children. Further prospective studies for myopia prediction in preschool children with longer follow up period and exploring the mechanisms underlying the relationship between AL and AL/CR and SE are needed.

## Data Availability

All data included in this study are available from the corresponding author upon reasonable request.

## Abbreviations

AL: Axial Length
CR: Corneal Radius of Curvature
SE: Spherical Equivalent
HR: Hazard Ratio
CR1: The greatest corneal radius of curvature
CR2: The lowest corneal radius of curvature
CP: Corneal Power

## References

[1] Holden BA, Fricke TR, Wilson DA, Jong M, Naidoo KS, Sankaridurg P, Wong TY, Naduvilath TJ, Resnikoff S (2016). Global prevalence of myopia and high myopia and temporal trends from 2000 through 2050. Ophthalmology.

[2] He M, Zeng J, Liu Y, Xu J, Pokharel GP, Ellwein LB (2004). Refractive error and visual impairment in urban children in southern China. Invest Ophthalmol Vis Sci.

[3] Dirani M, Zhou B, Hornbeak D, Chang BC, Gazzard G, Chia A, Ling Y, Selvaraj P, Young TL, Varma R (2010). Prevalence and causes of decreased visual acuity in Singaporean Chinese preschoolers. Br J Ophthalmol.

[4] Verkicharla PK, Ohno-Matsui K, Saw SM (2015). Current and predicted demographics of high myopia and an update of its associated pathological changes. Ophthalmic Physiol Opt.

[5] Saw SM, Tong L, Chua WH, Chia KS, Koh D, Tan DT, Katz J (2005). Incidence and progression of myopia in Singaporean school children. Invest Ophthalmol Vis Sci.

[6] Saw SM, Nieto FJ, Katz J, Schein OD, Levy B, Chew SJ (2000). Factors related to the progression of myopia in Singaporean children. Optom Vis Sci.

[7] Wolffsohn JS, Flitcroft DI, Gifford KL, Jong M, Jones L, Klaver CCW, Logan NS, Naidoo K, Resnikoff S, Sankaridurg P (2019). IMI - myopia control reports overview and introduction. Invest Ophthalmol Vis Sci.

[8] Zhang M, Gazzard G, Fu Z, Li L, Chen B, Saw SM, Congdon N (2011). Validating the accuracy of a model to predict the onset of myopia in children. Invest Ophthalmol Vis Sci.

[9] Borchert MS, Varma R, Cotter SA, Tarczy-Hornoch K, McKean-Cowdin R, Lin JH, Wen G, Azen SP, Torres M, Tielsch JM (2011). Risk factors for hyperopia and myopia in preschool children the multi-ethnic pediatric eye disease and Baltimore pediatric eye disease studies. Ophthalmology.

[10] Wolffsohn JS, Kollbaum PS, Berntsen DA, Atchison DA, Benavente A, Bradley A, Buckhurst H, Collins M, Fujikado T, Hiraoka T (2019). IMI - clinical myopia control trials and instrumentation report. Invest Ophthalmol Vis Sci.

[11] Sankaridurg P, He X, Naduvilath T, Lv M, Ho A, Smith E, Erickson P, Zhu J, Zou H, Xu X (2017). Comparison of noncycloplegic and cycloplegic autorefraction in categorizing refractive error data in children. Acta Ophthalmol.

[12] Brown NP, Koretz JF, Bron AJ (1999). The development and maintenance of emmetropia. Eye (Lond).

[13] Mutti DO, Mitchell GL, Jones LA, Friedman NE, Frane SL, Lin WK, Moeschberger ML, Zadnik K (2005). Axial growth and changes in lenticular and corneal power during emmetropization in infants. Invest Ophthalmol Vis Sci.

[14] Chua SY, Ikram MK, Tan CS, Lee YS, Ni Y, Shirong C, Gluckman PD, Chong YS, Yap F, Wong TY (2015). Relative contribution of risk factors for early-onset myopia in Young Asian children. Invest Ophthalmol Vis Sci.

[15] Scheiman M, Gwiazda J, Zhang Q, Deng L, Fern K, Manny RE, Weissberg E, Hyman L, Group C (2016). Longitudinal changes in corneal curvature and its relationship to axial length in the correction of myopia evaluation trial (COMET) cohort. J Optom.

[16] Zhao KK, Yang Y, Wang H, Li L, Wang ZY, Jiang F, Qu JF (2019). Axial length/corneal radius of curvature ratio and refractive development evaluation in 3- to 4-year-old children: the Shanghai Pudong eye study. Int J Ophthalmol.

[17] Borchert M, Wang Y, Tarczy-Hornoch K, Cotter S, Deneen J, Azen S, Varma R, Group MS (2008). Testability of the Retinomax autorefractor and IOLMaster in preschool children: the Multi-ethnic Pediatric Eye Disease Study. Ophthalmology.

[18] Guo X, Fu M, Ding X, Morgan IG, Zeng Y, He M (2017). Significant axial elongation with minimal change in refraction in 3- to 6-year-old Chinese preschoolers: the Shenzhen kindergarten eye study. Ophthalmology.

[19] Wang Z, Huang D, Chen X, Zhu H, Sun Q, Wang Y, Zhang X, Wang Y, Zhai L, Wang C (2019). Preschool children exhibit evident compensatory role of internal astigmatism in distribution of astigmatism: the Nanjing eye study. Invest Ophthalmol Vis Sci.

[20] Multi-Ethnic Pediatric Eye Disease Study G (2009). Prevalence and causes of visual impairment in African-American and Hispanic preschool children: the multi-ethnic pediatric eye disease study. Ophthalmology.

[21] Zhao X, Li R, Huang D, Tong H, Zhu H, Wang Y, Zhang X, Hao Q, Sun Q, Liu H (2020). Decreased retinal thickness in preschool offspring of maternal gestational hypertension: the Nanjing eye study. Acta Ophthalmol.

[22] Shen S, Li X, Li R, Huang D, Zhao X, Zhang X, Hao Q, Sun Q, Tong H, Zheng X (2021). Association of sleep disorders with subfoveal choroidal thickness in preschool children. Eye (Lond).

[23] Morgan IG, Rose KA, Ellwein LB (2010). Refractive error study in children survey G: is emmetropia the natural endpoint for human refractive development? An analysis of population-based data from the refractive error study in children (RESC). Acta Ophthalmol.

[24] Zhang L, He X, Qu X, You X, Wang B, Shi H, Tan H, Zou H, Zhu J (2018). Refraction and ocular biometry of preschool children in Shanghai, China. J Ophthalmol.

[25] Momeni-Moghaddam H, Hashemi H, Zarei-Ghanavati S, Ostadimoghaddam H, Yekta A, Khabazkhoob M (2019). Four-year change in ocular biometric components and refraction in schoolchildren: a cohort study. J Curr Ophthalmol.

[26] Saw SM, Carkeet A, Chia KS, Stone RA, Tan DT (2002). Component dependent risk factors for ocular parameters in Singapore Chinese children. Ophthalmology.

[27] Fan DS, Lai C, Lau HH, Cheung EY, Lam DS (2011). Change in vision disorders among Hong Kong preschoolers in 10 years. Clin Exp Ophthalmol.

[28] Yam JC, Tang SM, Kam KW, Chen LJ, Yu M, Law AK, Yip BH, Wang YM, Cheung CYL, Ng DSC (2020). High prevalence of myopia in children and their parents in Hong Kong Chinese population: the Hong Kong children eye study. Acta Ophthalmol.

[29] Chua SY, Sabanayagam C, Cheung YB, Chia A, Valenzuela RK, Tan D, Wong TY, Cheng CY, Saw SM (2016). Age of onset of myopia predicts risk of high myopia in later childhood in myopic Singapore children. Ophthalmic Physiol Opt.

[30] McCullough S, Adamson G, Breslin KMM, McClelland JF, Doyle L, Saunders KJ (2020). Axial growth and refractive change in white European children and young adults: predictive factors for myopia. Sci Rep.

[31] Mutti DO, Hayes JR, Mitchell GL, Jones LA, Moeschberger ML, Cotter SA, Kleinstein RN, Manny RE, Twelker JD, Zadnik K (2007). Refractive error, axial length, and relative peripheral refractive error before and after the onset of myopia. Invest Ophthalmol Vis Sci.

[32] Rozema J, Dankert S, Iribarren R, Lanca C, Saw SM (2019). Axial growth and Lens power loss at myopia onset in Singaporean children. Invest Ophthalmol Vis Sci.

[33] Zadnik K, Sinnott LT, Cotter SA, Jones-Jordan LA, Kleinstein RN, Manny RE, Twelker JD, Mutti DO (2015). Collaborative longitudinal evaluation of E, refractive error study G: prediction of juvenile-onset myopia. JAMA Ophthalmol.

[34] Tao Z, Deng H, Zhong H, Yu Y, Zhao J, Chen S, Li S, Zhu M (2021). A longitudinal study of the effect of ocular biometrics measures on myopia onset. Graefes Arch Clin Exp Ophthalmol.

[35] Jones-Jordan LA, Sinnott LT, Manny RE, Cotter SA, Kleinstein RN, Mutti DO, Twelker JD, Zadnik K (2010). Collaborative longitudinal evaluation of E, refractive error study G: early childhood refractive error and parental history of myopia as predictors of myopia. Invest Ophthalmol Vis Sci.

[36] Jones LA, Sinnott LT, Mutti DO, Mitchell GL, Moeschberger ML, Zadnik K (2007). Parental history of myopia, sports and outdoor activities, and future myopia. Invest Ophthalmol Vis Sci.

[37] Jiang X, Tarczy-Hornoch K, Cotter SA, Matsumura S, Mitchell P, Rose KA, Katz J, Saw SM, Varma R, Consortium P (2020). Association of Parental Myopia with Higher Risk of myopia among multiethnic children before school age. JAMA Ophthalmol.

[38] Ye S, Liu S, Li W, Wang Q, Xi W, Zhang X (2019). Associations between anthropometric indicators and both refraction and ocular biometrics in a cross-sectional study of Chinese schoolchildren. BMJ Open.

[39] Jonas JB, Ang M, Cho P, Guggenheim JA, He MG, Jong M, Logan NS, Liu M, Morgan I, Ohno-Matsui K (2021). IMI prevention of myopia and its progression. Invest Ophthalmol Vis Sci.

[40] Nemeth J, Tapaszto B, Aclimandos WA, Kestelyn P, Jonas JB, De Faber JHN, Januleviciene I, Grzybowski A, Nagy ZZ, Parssinen O (2021). Update and guidance on management of myopia. European Society of Ophthalmology in cooperation with International Myopia Institute. Eur J Ophthalmol.

